# Alterations in von Willebrand Factor Levels in Patients with Malaria: A Systematic Review and Meta-Analysis of Disease Severity

**DOI:** 10.3390/medicina61040767

**Published:** 2025-04-21

**Authors:** Suriyan Sukati, Rujikorn Rattanatham, Frederick Ramirez Masangkay, Ching-Ping Tseng, Manas Kotepui

**Affiliations:** 1 Medical Technology, School of Allied Health Sciences, Walailak University, Tha Sala, Nakhon Si Thammarat 80160, Thailand; suriyan.su@wu.ac.th; 2 Hematology and Transfusion Science Research Center, Walailak University, Tha Sala, Nakhon Si Thammarat 80160, Thailand; 3 Medical Technology Program, Faculty of Science, Nakhon Phanom University, Nakhon Phanom 48000, Thailand; 4 Department of Medical Technology, University of Santo Tomas, Manila 1008, Philippines; 5 Department of Medical Biotechnology and Laboratory Science, College of Medicine, Chang Gung University, Taoyuan 333, Taiwan

**Keywords:** malaria, *Plasmodium*, von Willebrand factor, vWF, meta-analysis

## Abstract

*Background and Objectives*: Elevated von Willebrand factor (vWF) levels have been reported in malaria, but their relationship with disease severity remains unclear. This study aimed to compare vWF levels between *Plasmodium*-infected and uninfected individuals and assess changes in severe infections. *Materials and Methods*: The systematic review was registered in PROSPERO (CRD42024558479). A comprehensive search across six databases identified studies reporting vWF levels in malaria. A meta-analysis was conducted using a random-effects model, with standardized mean difference (SMD) as the effect measure due to varying measurement units. Heterogeneity was assessed using the *I*^2^ statistic. *Results*: Of 1647 identified records, 26 studies met the inclusion criteria. The meta-analysis showed significantly higher vWF levels in *Plasmodium*-infected individuals compared to uninfected controls (*p* < 0.001, SMD: 2.689 [95% CI 1.362; 4.017], *I*^2^: 98.1%, 12 studies, 3109 participants). However, no significant difference was found between severe and less severe cases (*p* = 0.051, SMD: 3.551 [95% CI −0.007; 7.109], *I*^2^: 99.3%, 8 studies, 1453 participants). *Conclusions*: vWF levels are significantly elevated in individuals with *Plasmodium* infections, indicating a potential role in malaria pathophysiology. Although levels tend to be higher in severe cases, current evidence is insufficient to support vWF as a reliable marker for disease severity. Further prospective and well-controlled studies are needed to validate its diagnostic and prognostic value in malaria management.

## 1. Introduction

Malaria is caused by at least one of the five *Plasmodium* species that infect humans, including *Plasmodium falciparum*, *P. vivax*, *P. malariae*, *P. ovale*, and *P. knowlesi* [1]. Malaria infection begins when a female *Anopheles* mosquito carrying *Plasmodium* sporozoites bites a human, injecting these parasites directly into the bloodstream [2]. The sporozoites then migrate to the liver, invade liver cells, and replicate during the liver stage. Subsequently, they are released into the bloodstream as merozoites, invading red blood cells, multiplying within them, and causing their destruction, leading to the onset of malaria symptoms [3]. In 2022, there were 249 million reported cases worldwide, reflecting a significant increase of five million cases from the previous year, and the disease resulted in approximately 608,000 deaths [4].

The spectrum of malaria symptoms ranges from asymptomatic cases to mild/uncomplicated cases and even severe/complicated disease [5]. Uncomplicated or mild malaria generally presents with fever, chills, sweating, headaches, vomiting, and anemia. In contrast, severe or complicated malaria can lead to serious complications, including unrousable coma, respiratory distress, severe anemia, acute kidney injury, and multi-organ dysfunction [5]. *Plasmodium falciparum* is particularly notorious for causing severe complications such as cerebral malaria, which can escalate to coma or death. This severe form occurs when infected red blood cells adhere to endothelial cells, the inner lining of blood vessels, leading to blockages, reduced blood flow, and inflammation in the brain [6]. Furthermore, the disease also significantly affects blood coagulation and vascular function [7,8]. During infection, endothelial cells become activated or damaged, increasing the expression of adhesion molecules and pro-inflammatory factors [9]. This activation results in a pro-thrombotic state where platelets become excessively activated and aggregated, leading to microclot formation. Consequently, this elevated platelet activity, combined with the ongoing coagulation cascade, increases the risk of thrombotic complications, including disseminated intravascular coagulation (DIC) [10,11].

Von Willebrand factor (vWF) is a crucial glycoprotein involved in hemostasis, synthesized primarily in endothelial cells and megakaryocytes, which are the precursors to platelets [12]. Within platelets, vWF is stored in alpha granules and released upon activation. Its main functions include facilitating platelet adhesion to sites of vascular injury and aiding in blood clot formation [13]. The vWF exists as large multimers that are cleaved into smaller units by the enzyme ADAMTS13, a process essential for regulating vWF activity and ensuring proper blood clotting [14]. One of the critical roles of vWF is stabilizing clotting factor VIII, protecting it from rapid degradation, and thereby aiding in the formation of stable blood clots [14]. vWF levels are measured through blood tests to assess their concentration and functionality, which is crucial for diagnosing bleeding disorders and evaluating hemostasis [15].

Elevated levels of vWF are observed in various conditions, including inflammatory disorders, stress responses, and certain cancers [16,17,18], and can lead to excessive platelet aggregation and increased thrombotic activity [19]. Infections, particularly those that induce systemic inflammation, can alter vWF levels, potentially contributing to disease complications. Malaria, caused by *Plasmodium* spp., is a major global health burden, with severe cases often leading to vascular dysfunction and coagulopathy. Several studies have suggested that vWF levels are elevated in malaria [20,21,22,23]; however, the nature of this association, particularly concerning disease severity, remains unclear. While some reports indicate a significant increase in vWF levels in infected individuals, the extent to which vWF contributes to malaria pathophysiology or serves as a biomarker for disease severity has not been systematically assessed.

Given the inconsistent findings in the literature and the potential role of vWF in malaria-associated coagulopathies, a comprehensive synthesis of existing evidence is necessary. This systematic review and meta-analysis aimed to consolidate data on vWF levels in malaria and evaluate their association with disease severity. By providing a clearer understanding of vWF alterations in malaria, this study may help inform clinical management strategies, improve early detection of hemostatic imbalances, and guide future research on the prognostic value of vWF in malaria outcomes.

## 2. Materials and Methods

### 2.1. Protocol and Registration

This systematic review’s meta-analysis protocol was registered at the International Prospective Register of Systematic Reviews (PROSPERO, CRD42024558479). The systematic review adhered to the Preferred Reporting Items for Systematic Reviews and Meta-Analyses (PRISMA) guideline [24].

### 2.2. Definitions

The severe case of *P. falciparum* infection is defined by the presence of asexual parasitemia from *P. falciparum*, accompanied by one or more of the following complications: hypoglycemia, prostration, hyperparasitemia, acidosis, impaired consciousness, renal impairment, pulmonary edema, significant bleeding, shock, multiple convulsions, or severe malarial anemia. Severe vivax and knowlesi malaria are defined in a manner similar to falciparum malaria, but without specific parasite density thresholds [25]. Non-severe malaria, also referred to as less severe cases, is characterized by malaria parasitemia without the complications associated with severe malaria [26,27]. It includes uncomplicated malaria and asymptomatic *Plasmodium* infections.

### 2.3. Systematic Review Question

The systematic review question was developed using the PECO framework as described previously [28,29]. The target population (P) included individuals residing in malaria-endemic regions; the exposure of interest (E) was *Plasmodium* infection, including severe or less severe cases; the comparison group (C) included participants without *Plasmodium* infection or those without less severe cases; and the measured outcome (O) was the level of vWF. The primary question addressed the difference in vWF levels between participants with *Plasmodium* infection and those without infection. The secondary question examined the changes in vWF levels in participants with severe *Plasmodium* infections compared to those with less severe infections.

### 2.4. Database Searches

Comprehensive searches for relevant articles reporting vWF in human malaria cases were conducted across six major databases: EMBASE, MEDLINE, Ovid, Scopus, PubMed, and ProQuest. The general search strategy used was: “(“Von Willebrand factor” OR vWF OR “Factor VIIIR-Ag” OR “Factor VIIIR Ag” OR “Factor VIIIR-RCo” OR “Factor VIIIR RCo” OR “Factor VIII-Related Antigen” OR “Factor VIII Related Antigen” OR “Ristocetin-Willebrand Factor” OR “Ristocetin Willebrand Factor” OR “von Willebrand Protein” OR “Ristocetin Cofactor” OR “Plasma Factor VIII Complex”) AND (malaria OR *Plasmodium* OR “*Plasmodium* Infection” OR “Remittent Fever” OR “Marsh Fever” OR Paludism)”. The search strategies in other databases varied slightly (Table S1). The searches were performed from inception to 13 June 2024 without publication date or language restrictions. Searches in Google Scholar were conducted to identify studies that were not indexed in the major databases.

### 2.5. Eligibility Criteria

The eligibility criteria for the study focused on selecting human studies that examined the relationship between malaria and vWF. Only cross-sectional, cohort, and case-control studies were included. Studies had to provide relevant data on vWF levels in individuals with *Plasmodium* infections, focusing on severe malaria. Exclusion criteria included in-vitro or animal experiments, reviews/systematic reviews/meta-analyses, case reports/series, commentaries/opinions/letters, and studies without data on vWF levels in participants with *Plasmodium* infections.

### 2.6. Study Selection and Data Extraction

After retrieving studies from each database, duplicates were removed using automated methods, followed by manual removal (Endnote version 20.0, Clarivate, Philadelphia, PA, USA). The titles and abstracts of the remaining articles were independently screened for relevance. After reviewing their full texts against the eligibility criteria, potentially eligible articles were assessed. Articles not meeting the criteria were excluded, and specific reasons were provided. Studies that fulfilled the eligibility criteria were included for data extraction and risk of bias assessment. Data from the selected studies were extracted based on their characteristics, including publication year, geographic location, study design, participant demographics, diagnostic methods for malaria and vWF, and the type of blood samples used for measuring vWF levels (plasma or serum). Two authors (SS and MK) independently conducted the study selection and data extraction. Any disagreements were resolved through discussion with a third author (RR) until a consensus was reached.

### 2.7. Risk of Bias Assessment

The Joanna Briggs Institute (JBI) critical appraisal checklists were used to assess the potential bias in the included studies [30]. Cross-sectional studies were evaluated based on the clear definition of inclusion criteria, consistent measurement of exposure and outcome assessments, identification and control of confounding factors, and the appropriateness of statistical analysis. Case-control studies were appraised for the selection of cases and controls, identification of confounding factors, consistency and accuracy in exposure measurement, and the appropriateness of statistical analysis. Cohort studies were assessed for the appropriateness of participant selection, the identification and management of confounding factors, the accuracy of outcome measurements, the sufficiency and consistency of follow-up periods between groups, and the appropriateness of statistical analysis. Two authors (SS and RR) independently assessed the risk of bias in selected studies. Disagreements were settled through a third author (MK) for the final inclusion.

### 2.8. Data Synthesis

The protocol for data synthesis followed previous studies [26,29]. A narrative synthesis was employed to describe the changes in vWF levels among participants with *Plasmodium* infections compared to those without, and between participants with severe and non-severe forms of the infection. The meta-analysis was performed to synthesize the quantitative data on vWF levels in these groups. The random-effects model was applied to account for heterogeneity among the included studies [26,31]. The standardized mean difference (SMD), also known as Cohen’s effect size, was chosen as the effect measure because the included studies reported vWF levels using different measurement units and methods [32]. SMD allows for pooling results across studies with varying scales by standardizing the effect size, making comparisons more meaningful. The interpretation of SMD is as follows: SMD = 0.2 indicates a small effect, SMD = 0.5 a moderate effect, and SMD = 0.8 a large effect [32]. A fixed-effects model was also conducted in parallel as part of a sensitivity analysis to ensure consistency of results across both models. Heterogeneity was assessed using the inconsistency index (*I*^2^), with *I*^2^ values greater than 50% indicating significant variability among studies [33]. Potential sources of heterogeneity were explored through meta-regression and subgroup analyses, considering factors such as publication year, study design, geographic location, participant age, *Plasmodium* species, diagnostic methods for malaria and vWF, and blood sample type for vWF measurement. An influence analysis was performed to assess whether excluding any single study would affect the meta-analysis results [34]. Publication bias was evaluated through the funnel plot asymmetry and Egger’s regression test when at least ten studies were included in the meta-analysis [27,35]. Statistical analyses were performed using RStudio (Version: 2024.04.2+764) [36], with a *p*-value less than 0.05 considered statistically significant.

## 3. Results

### 3.1. Search Results

**Figure 1:**
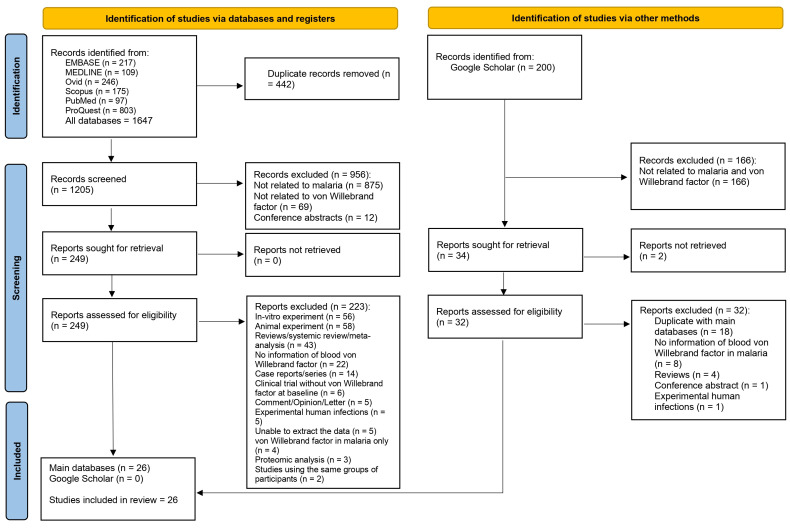
The PRISMA 2020 flow diagram illustrates the step-by-step process of selecting the studies in the systematic review.

Initially, 1647 articles were retrieved from six databases. Following the removal of 442 duplicates, 1205 records were screened, and 956 were excluded due to irrelevance to the study topic (malaria and von Willebrand factor). A total of 249 reports were sought for retrieval, and none were missing. Following an eligibility assessment, 223 reports were excluded for reasons such as animal studies, in-vitro studies, reviews, and lack of relevant data. Finally, 26 studies were incorporated into the systematic review. Additionally, after reviewing 200 records retrieved from Google Scholar, 34 articles were selected for retrieval. Of these 34 records, 32 underwent eligibility assessment, and all were excluded as duplicates of studies included from the main databases or for other specific reasons (Figure 1).

### 3.2. Key Characteristics of Included Studies

Most studies (50%) were published from 2010 to 2019, with 30.77% from 2020–2024 and 19.23% from 2000–2009. Cross-sectional studies comprised most of the study design (46.15%), followed by cohort (38.46%) and case-control studies (15.38%). The majority of studies were performed in Africa (69.23%). A smaller number of studies occurred in Asia (19.23%) and South America (11.54%). The primary focus of the included studies was on *P. falciparum* infections (69.23%). Half of the studies (50%) involved children as participants, while 23.08% focused on adults. For detecting *Plasmodium*, 42.31% of studies used microscopic methods, while others used combinations of microscopy, rapid diagnostic tests (RDT), and PCR. The vWF was primarily measured using ELISA (73.08%), and most studies analyzed plasma samples (88.46%) (Table 1). Details of all studies included in the present study are listed in Table S2.

### 3.3. Risk of Bias

All cross-sectional studies clearly defined their inclusion criteria and provided detailed descriptions of the study subjects and settings. The exposure was consistently measured validly and reliably across studies, with standard criteria applied for condition measurement. However, confounding factors were not always identified or addressed. All studies used valid outcome measures and appropriate statistical analyses. All case-control studies met most of the criteria except for one study that was unclear about the validity and reliability of the exposure [37]. The exposure measurement methods were consistent across both cases and controls, with all studies identifying and addressing confounding factors using appropriate strategies. Outcome assessments were valid; all studies used a sufficient exposure period and conducted appropriate statistical analyses. All cohort studies ensured that groups were similar and recruited from the same population, with exposure measured similarly across both exposed and unexposed groups. However, some studies did not identify or address confounding factors [38,39]. Other studies performed well across all criteria, including handling incomplete follow-up and conducting reliable statistical analysis (Table S3).

### 3.4. vWF Levels Between Plasmodium-Infected and -Uninfected Controls

Twenty-four studies in total examined the differences in vWF levels between individuals with *Plasmodium* infections and non-malarial controls [20,21,22,23,37,38,39,40,41,42,43,44,45,46,47,48,49,50,51,52,53,54,55,56]. The majority of these studies reported a significant increase in vWF levels in *Plasmodium*-infected individuals compared to uninfected controls [21,22,23,41,42,43,44,45,47,50,54,55]. However, some studies found no significant difference between the two groups [37,40,46,48]. One study found no significant difference in vWF levels between adults with *Plasmodium* infections and non-malarial controls, but vWF levels were significantly increased in *Plasmodium*-infected children compared to uninfected children [39]. Additionally, vWF levels were significantly increased in cases of severe *Plasmodium* infections (including cerebral and uncomplicated malaria) compared to healthy controls, but there was no difference in vWF levels between cases of uncomplicated malaria and non-malarial febrile illnesses [49]. vWF levels were also significantly elevated in mild malaria compared to non-malarial controls (non-malarial febrile illness and non-febrile illness) and in cerebral malaria compared to these same controls [53]. Similarly, vWF levels were significantly elevated in patients with both severe and non-severe malaria compared to controls [38]. Moreover, vWF levels were significantly increased in both uncomplicated malaria and asymptomatic *Plasmodium* infections compared to children without parasitemia [52]. Additionally, vWF levels were significantly elevated in severe *Plasmodium* infections compared to healthy individuals [20,51].

**Figure 2:**
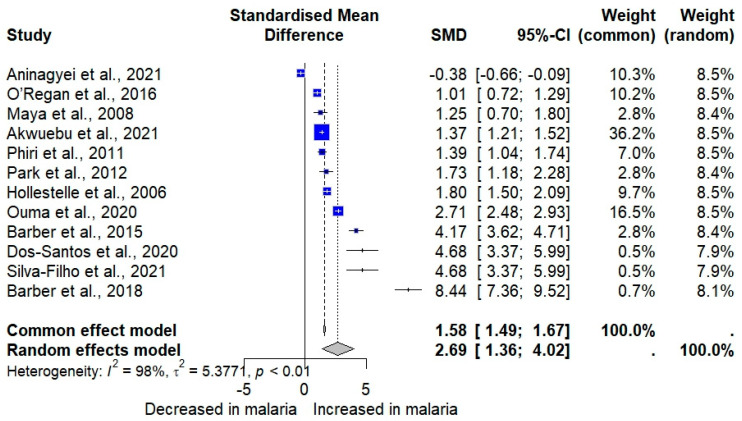
Forest plot showing the standardized mean differences (SMD) with 95% confidence intervals (CI) for von Willebrand Factor (vWF) levels in patients with *Plasmodium* infections versus those without infections. Each horizontal line represents an individual study, with the square indicating the point estimate of the SMD and the square’s size reflecting the study’s weight in the meta-analysis. The diamond at the bottom represents the overall effect estimate from both the common/fixed-effect model (SMD = 1.58, 95% CI [1.49, 1.67]) and the random-effects model (SMD = 2.69, 95% CI [1.36, 4.02]). The analysis indicates significant heterogeneity among studies (*I*^2^ = 98%, *p* < 0.01). Values greater than 0 on the x-axis indicate an increased vWF in patients with malaria, while values less than 0 indicate a decreased vWF in patients with malaria [23,38,40,42,44,48,49,51,52,53,55,56].

**Figure 3:**
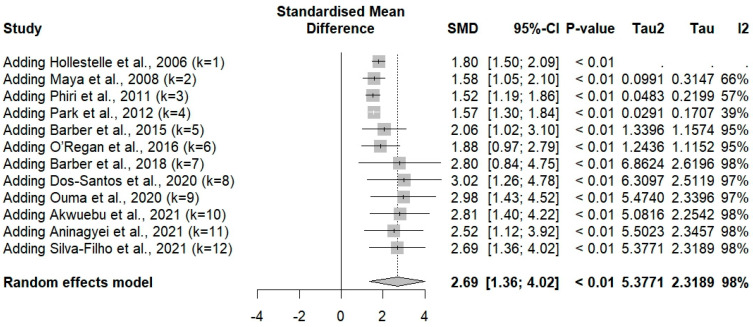
Cumulative meta-analysis using a random-effects model showing the standardized mean difference (SMD) and 95% confidence intervals (CI) for von Willebrand Factor (vWF) levels in patients with *Plasmodium* infections versus those without infections. Each row represents the sequential addition of individual studies (k = 1 to k = 12) to the cumulative analysis. The SMD values and their corresponding 95% CIs are displayed for each step. The overall cumulative effect size is shown at the bottom (SMD: 2.69 [95% CI: 1.36, 4.02], *p* < 0.01). The degree of heterogeneity among the included studies is indicated by Tau^2^, Tau, and *I*^2^ values. The *I*^2^ value of 98% suggests substantial heterogeneity across the studies [23,38,40,42,44,48,49,51,52,53,55,56].

The meta-analysis using quantitative data (mean/median, standard deviation/range) of vWF showed that vWF levels were significantly increased in participants with *Plasmodium* infections compared to those without infections (*p* < 0.001, SMD: 2.6894 [95% CI 1.3623; 4.0165], *I*^2^: 98.1%, number of participants: 3109, random-effects model; Figure 2). Similarly, the meta-analysis using the fixed-effects model demonstrated a significant increase in vWF levels in patients with *Plasmodium* infections compared to those without infections (*p* < 0.001, SMD: 1.5831 [95% CI 1.4914; 1.6748]; Figure 2). The high heterogeneity (*I*^2^ > 98%) indicates substantial variability between studies. Therefore, the random-effects model is more appropriate and should be given greater interpretive weight in this meta-analysis. The cumulative meta-analysis shows a trend toward an increasing effect size (higher SMD values) over time (Figure 3).

The subgroup analysis showed significant differences in the SMDs of vWF levels associated with *Plasmodium* infections based on geographic location, age ranges, *Plasmodium* species, and diagnostic methods for malaria and vWF (Table 2). Notably, studies from Asia show a higher SMD (6.2783) compared to those conducted in South America (SMD: 4.6809) and Africa (SMD: 1.3580). Among the countries, Malaysian studies reported the highest SMD (6.2783), followed by Brazilian studies (SMD: 4.6809). Studies enrolling participants of all age ranges (children and adults) demonstrated a higher SMD (4.5980) than those enrolling children only (SMD: 1.6678) or adults only (SMD: 2.1086). There was also variability in SMD across different *Plasmodium* species. Studies involving participants infected with *P. vivax* showed a higher SMD (4.6809) compared to those with *P. falciparum* (SMD: 1.3580). Regarding diagnostic methods for malaria, studies using microscopy/PCR methods to detect malaria parasites exhibited a higher SMD (4.3629) than those using microscopy/RDT (SMD: 0.6690) or microscopy alone (SMD: 1.4942).

**Figure 4:**
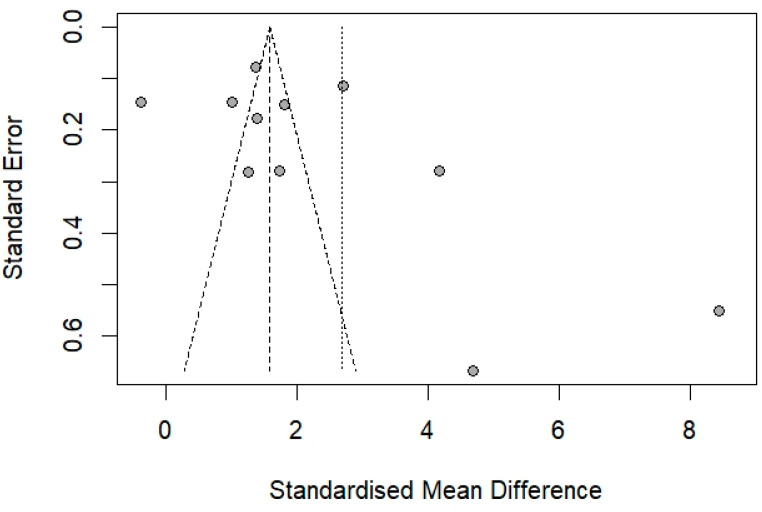
Funnel plot for the meta-analysis of von Willebrand factor (vWF) levels in participants with and without *Plasmodium* infections. The funnel plot depicts the relationship between the standardized mean differences (SMDs) of vWF levels and their standard errors from studies comparing *Plasmodium*-infected participants to uninfected participants. Each point represents a study included in the meta-analysis. The dashed lines represent the 95% confidence limits, and the vertical solid line indicates the overall pooled effect size. Asymmetry in the funnel plot suggests potential publication bias or small-study effects.

The influential analysis shows that the overall effect size (SMD) for vWF levels remains robust and statistically significant even when each study was omitted (Supplementary Table S1). This indicated that the findings of elevated vWF levels in *Plasmodium*-infected participants are consistent and not disproportionately affected by any single study. The funnel plot demonstrated asymmetry, suggesting potential publication bias or small-study effects (Figure 4). However, Egger’s test did not provide statistically significant evidence of publication bias (*p* = 0.1332).

### 3.5. vWF in Severe and Non-Severe Plasmodium Infections

Ten studies investigated the differences in vWF levels in participants with severe and non-severe *Plasmodium* infections [23,38,42,47,52,53,54,56,57,58]. vWF levels were significantly increased in participants with severe *Plasmodium* infections compared to those with mild or uncomplicated *Plasmodium* infections in several studies [47,52,53,54,56,57,58]. However, one study reported no significant difference in vWF levels between severe malaria (cerebral and non-cerebral) and mild malaria [23]. No significant alteration in vWF between participants with severe *Plasmodium* infections and those with non-severe *P. falciparum*, *P. vivax*, and *P. knowlesi* infections was found [38,42].

**Figure 5:**
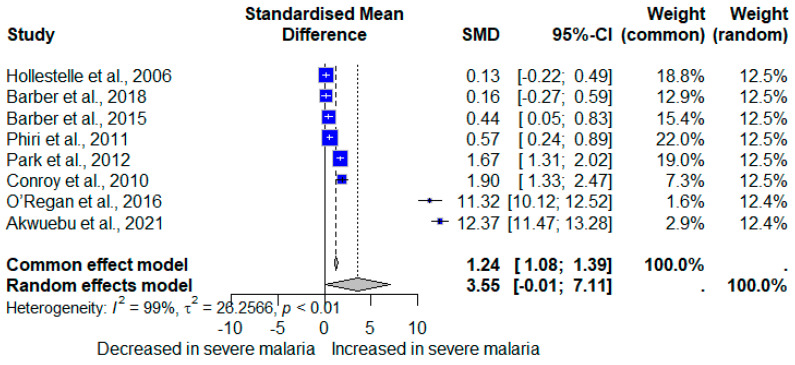
Forest plot showing the standardized mean differences (SMD) with 95% confidence intervals (CI) for von Willebrand Factor (vWF) levels in participants with severe and less severe *Plasmodium* infections. Each horizontal line represents an individual study, with the square indicating the point estimate of the SMD and the square’s size reflecting the study’s weight in the meta-analysis. The diamond at the bottom represents the overall effect estimate from both the common/fixed-effect model (SMD = 1.24, 95% CI [1.08, 1.39]) and the random-effects model (SMD = 3.55, 95% CI [−0.01, 7.11]). The analysis indicates significant heterogeneity among studies (*I*^2^ = 99%, *p* < 0.01). Values greater than 0 on the x-axis indicate an increased vWF in patients with severe malaria, while values less than 0 indicate a decreased vWF in patients with severe malaria [23,38,42,49,52,53,56,57].

**Figure 6:**
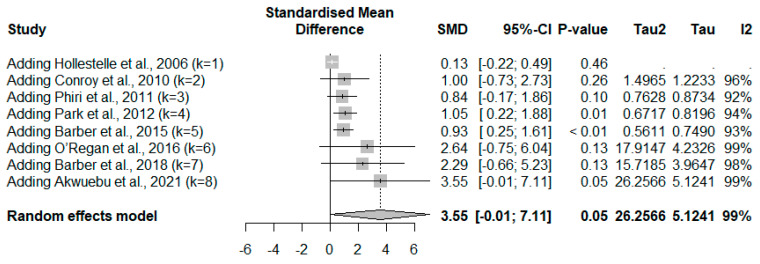
Cumulative meta-analysis using a random-effects model showing the standardized mean difference (SMD) and 95% confidence intervals (CI) for von Willebrand Factor (vWF) levels in participants with severe and less severe *Plasmodium* infections. Each row represents the sequential addition of individual studies (k = 1 to k = 8) to the cumulative analysis. The SMD values and their corresponding 95% CIs are displayed for each step. The overall cumulative effect size is shown at the bottom (SMD: 3.55 [95% CI: −0.01, 7.11], *p*: 0.05). The degree of heterogeneity among the included studies is indicated by Tau^2^, Tau, and *I*^2^ values. The *I*^2^ value of 99% suggests substantial heterogeneity across the studies [23,38,42,49,52,53,56,57].

The meta-analysis using quantitative data (mean/median, standard deviation/range) of vWF demonstrated no significant difference in vWF levels between severe compared to less severe cases (*p*: 0.0505, SMD: 3.5506 [95% CI −0.0074; 7.1085], *I*^2^: 99.3%, number of participants: 1453, random-effects model; Figure 5). Contrarily, the meta-analysis using the fixed-effects model demonstrated a significant increase in vWF levels in participants with severe compared to less severe *Plasmodium* infections (*p* < 0.001, SMD: 1.2384 [95% CI 1.0843; 1.3925]; Figure 5). The high heterogeneity (*I*^2^ > 99%) indicates substantial variability between studies. Therefore, the random-effects model is more appropriate and should be given greater interpretive weight in this meta-analysis. The cumulative meta-analysis shows a trend toward an increasing effect size (higher SMD values) over time (Figure 6).

The subgroup analysis showed significant differences in the SMD of vWF levels related to severe *Plasmodium* infections based on publication years, study designs, and countries (Table 3). Notably, studies published between 2020 and 2024 showed a higher SMD (12.3712) compared to those published between 2010 and 2019 (SMD: 2.6492) and between 2000 and 2009 (SMD: 0.1348). Among the different study designs, cross-sectional studies reported a higher SMD (6.7968) than cohort studies (SMD: 0.3398). Regarding country-specific differences, studies conducted in Nigeria reported the highest SMD (12.3712), followed by those conducted in Malawi (SMD: 4.5730). No significant differences were observed in the subgroup analyses based on continents, *Plasmodium* species, age ranges, diagnostic methods for malaria, vWF quantification methods, or the type of blood samples used for vWF measurement.

The influential analysis suggested that certain studies [23,38,42] have a substantial impact on the pooled effect size. Their exclusion increased the SMD, leading to marginally significant *p*-values (Supplementary Table S2). The influence of individual studies on the statistical significance of the results indicated that the findings are sensitive to including specific studies. The funnel plot and Egger’s test were not performed because the meta-analysis included fewer than ten studies.

### 3.6. vWF in Mortality, Complications, Plasmodium Species, and Parasite Density

Concerning mortality, vWF levels were linked to mortality and gradually decreased over time in survivors of severe malaria [22]. However, no difference in vWF levels was noted between children who survived severe malaria and those who subsequently died [58]. In studies comparing different forms of severe malaria, there was no significant difference in vWF levels between cerebral and non-cerebral severe malaria [23]. However, vWF levels were significantly higher in cerebral malaria compared to severe malarial anemia, and they increased with the severity of acute kidney failure [51]. Similarly, no significant difference in vWF levels was observed between cerebral malaria patients with retinopathy and those without retinopathy [53,57]. For different *Plasmodium* species, no significant difference in vWF antigen concentrations was observed between *P. vivax* and *P. falciparum* infections. However, peripheral parasitemia was correlated with vWF levels in both types of malaria [38,43]. Moreover, vWF levels were significantly higher in *P. vivax* infections with high total parasite biomass compared to *P. vivax* with low total parasite biomass and healthy controls [55]. In patients with malaria and HIV co-infection, vWF levels were more profoundly elevated compared to those with either condition alone, and levels were significantly increased in both malaria and HIV patients compared to those with only one of these infections [45,50].

## 4. Discussion

The study’s results demonstrated a significant alteration in vWF levels in individuals with *Plasmodium* infections, with most studies reporting an increase in vWF levels compared to those without the infection. The meta-analysis findings reinforce this observation, showing a statistically significant increase in vWF levels among *Plasmodium*-infected individuals. This elevation of vWF could serve as a key biomarker of endothelial activation, reflecting the inflammatory and coagulation disturbances caused by malaria.

The mechanism underlying this increase may involve infected red blood cells releasing microvesicles that stimulate pro-inflammatory cytokines, such as interleukin 12 (IL-12), IL-6, and tumor necrosis factor (TNF) [59,60]. TNF may then activate endothelial cells by promoting the expression of adhesion molecules like intercellular adhesion molecule 1 (ICAM-1), vascular cell adhesion molecule 1 (VCAM-1), and the secretion of chemokines that attract immune cells to the site of infection [61,62]. These activated endothelial cells increase the synthesis and release of circulating vWF from Weibel–Palade bodies, promoting platelet adhesion and aggregation, which leads to a hypercoagulable state. The presence of ultra-large vWF multimers further enhances platelet binding [20].

Moreover, elevated vWF may be linked to the decrease in platelet count, which was notably reduced in malaria patients, especially those with severe malaria [63,64]. Importantly, vWF serves as a marker of acute endothelial activation in the early phase of *Plasmodium* infection [23]. Additionally, the highest vWF levels were detected in patients with severe malaria, including cerebral and non-cerebral manifestations [23]. There was a strong association between vWF and activated vWF with platelet count. Increased vWF levels proportionally accompanied decreased platelets [65]. This may suggest that endothelial activation drives vWF release, facilitating platelet adhesion to the vascular wall and contributing to platelet depletion in severe malaria, where thrombocytopenia and microvascular obstruction are common complications [53]. In addition, several other markers are activated and released during endothelial activation, including ICAM-1, VCAM-1, E-selectin, and various cytokines and chemokines [65,66]. These factors further highlight the complex interplay between inflammation, coagulation, and immune response in malaria infections.

Subgroup analysis revealed geographic differences in vWF levels. Studies from Asia, particularly Malaysia, showed the highest SMD (6.2783), followed by South America (Brazil) and Africa. This variability may reflect differences in the *Plasmodium* species endemic to each region. For instance, *P. knowlesi* infections are predominantly observed in Southeast Asian countries, especially Malaysia, where macaque monkeys live near human populations [67]. In South America, particularly Brazil, *P. vivax* is the most prevalent *Plasmodium* species [68]. Meanwhile, *P. falciparum* is endemic in sub-Saharan Africa [69]. The subgroup analyses revealed that vWF levels vary significantly across different *Plasmodium* species. While vWF levels are elevated across *Plasmodium* species, the extent and potential clinical relevance vary, with *P. falciparum* and *P. vivax* showing the most consistent associations. In comparisons between infected and uninfected individuals, *P. vivax* and *P. knowlesi* infections are associated with markedly elevated vWF levels that may suggest a strong inflammatory or endothelial activation response for these *Plasmodium* species. *P. falciparum* infections show a moderate increase in vWF levels, though this is based on a larger number of studies and accompanied by high heterogeneity. In analyses comparing severe and less severe malaria, vWF levels are significantly elevated in severe *P. falciparum* cases, suggesting a potential role of vWF in disease severity. Conversely, *P. knowlesi* and mixed-species infections (e.g., *P. falciparum* and *P. vivax*) show smaller or non-significant differences in vWF levels between severity groups.

High vWF levels in patients with *P. knowlesi* infections may reflect coagulation disturbances. A previous systematic review showed that *P. knowlesi* infections present more abnormal bleeding cases than *P. falciparum* infections [70]. Therefore, the consumption of coagulation factors in *P. knowlesi* infections may occur more frequently than in infections with other *Plasmodium* species, which could explain the elevated levels of fibrin degradation products in these patients. Additionally, environmental and genetic factors may influence the endothelial responses to these infections, further contributing to the observed geographic variability in vWF levels.

For the association between vWF levels and severe malaria, while most studies report elevated vWF levels in severe cases, the meta-analysis found no significant difference between severe and non-severe cases using the random-effects model. However, the fixed-effects model did show the opposite result. The different results of both models may be explained by the high heterogeneity (*I*^2^ = 99.3%) across studies, suggesting that the relationship between vWF levels and malaria severity is complex and influenced by factors such as the year of the study, study design, and geographical location, as noted in the subgroup analysis. Current evidence shows elevated vWF levels in uncomplicated malaria and asymptomatic *Plasmodium* infections, indicating that increased vWF might not be exclusive to severe cases but could serve as a general marker of *Plasmodium* infection.

Additionally, the observed rise in vWF levels during *Plasmodium* infections has significant implications for understanding the pathophysiology of malaria-related complications. The increased level of vWF indicates endothelial activation and may also serve as a potential biomarker for monitoring disease progression and treatment response. Since vWF is involved in both hemostasis and inflammation, its elevated levels in malaria may reflect the connection between the immune response and coagulation pathways during infection [71]. This suggests that vWF could play a key role in the thrombotic and hemorrhagic manifestations often seen in severe malaria cases [11,22]. Therefore, measuring vWF levels may offer additional insights for clinicians assessing patient risk and guiding therapeutic decisions. However, the practicality of implementing vWF testing in routine clinical settings, particularly in resource-limited areas, remains challenging due to limited access to specialized assays. Moreover, the infecting *Plasmodium* species may affect the clinical relevance of vWF levels, which is not always identifiable through routine microscopy without molecular testing.

The observed trend toward increasing effect sizes in vWF levels over time, as shown in the cumulative meta-analyses—both in *Plasmodium*-infected individuals compared to uninfected controls and in severe compared to less severe malaria cases—may be influenced by advancements in laboratory techniques and increased standardization of vWF quantification methods (e.g., improved ELISA sensitivity and assay calibration) in recent years. Additionally, geographical differences in study populations over time may contribute, as variations in malaria transmission intensity, endemicity, and host immune responses can affect disease presentation and associated biomarkers such as vWF. The result of this study aligns with previous systematic reviews and meta-analyses, which found elevated vWF levels in COVID-19 patients, suggesting a link between vWF and the development of thrombosis in COVID-19 patients [72]. Additionally, vWF levels were significantly higher in COVID-19 patients with unfavorable outcomes [73,74]. A prior study also showed the association between high circulating vWF and adverse clinical outcomes in coronary artery disease patients [75]. Moreover, vWF was reported as a prognostic marker for cardiovascular complications in type 2 diabetes patients, as indicated by another systematic review and meta-analysis [76]. Elevated vWF levels were also linked to major adverse cardiac events, suggesting a prognostic role for higher vWF levels in atrial fibrillation patients [77].

The study has certain limitations. The heterogeneity of results from individual studies may affect the interpretation of the meta-analysis findings. The high degree of heterogeneity observed across studies may be due to variations in study design, population demographics (e.g., age range, endemicity), *Plasmodium* species, diagnostic methods, and assays used for measuring vWF. These differences limit the generalizability of the findings of the meta-analyses. The potential for publication bias in the meta-analysis comparing vWF levels between severe and non-severe cases was not assessed because fewer than ten studies were included. Despite these limitations, the study highlights the need for further investigation into the role of vWF in malaria pathogenesis. Future studies should aim to determine the clinical utility of vWF as a diagnostic or prognostic marker for *Plasmodium* infections. Although not sufficient on its own, elevated vWF levels may be regarded as a necessary condition that allows clinicians to suspect a potentially severe disease outcome, even if severe symptoms are not yet overt. This could contribute to earlier clinical suspicion and intervention, ultimately improving outcomes in patients with malaria.

## 5. Conclusions

This systematic review and meta-analysis indicate that vWF levels are significantly elevated in individuals with *Plasmodium* infections, suggesting a possible association with malaria pathophysiology. While vWF levels appear higher in severe cases, the evidence is insufficient to establish its utility as a reliable diagnostic or prognostic marker. The observed trends highlight the potential of vWF as a nonspecific marker of infection; however, its role in distinguishing between severe and non-severe malaria remains unclear. Further prospective and well-controlled studies are essential to clarify its diagnostic and prognostic value and to determine whether vWF can meaningfully contribute to clinical decision-making in malaria management.

## Figures and Tables

**Table 1:** Key characteristics of included studies (N = 26).

Key Characteristics	Number of Studies (N)	%
**Publication year**		
2000–2009	5	19.23
2010–2019	13	50.00
2020–2024	8	30.77
**Study designs**		
Cross-sectional study	12	46.15
Cohort study	10	38.46
Case-control study	4	15.38
**Study areas**		
Asia	5	19.23
Indonesia	2	7.69
Malaysia	2	7.69
Bangladesh	1	3.85
Africa	18	69.23
Ghana	4	15.38
Malawi	4	15.38
Uganda	4	15.38
Gabon	2	7.69
Nigeria	2	7.69
Rwanda	1	3.85
Mozambique	1	3.85
South America	3	11.54
Brazil	2	7.69
Colombia	1	3.85
***Plasmodium* species**		
*P. falciparum*	19	73.08
*P. vivax*	3	11.54
*P. falciparum, P. vivax*	2	7.69
*P. knowlesi*	1	3.85
*P. falciparum*, *P. vivax*, mixed infections, unclassified species	1	3.85
**Participants**		
Children	13	50.00
Adults	6	23.08
Children and adults	5	19.23
Not specified	2	7.69
**Methods for detecting *Plasmodium***		
Microscopic method	11	42.31
Microscopic method/RDT	8	30.77
Microscopic method/RDT	4	15.38
Microscopic method/RDT/PCR	2	7.69
RDT/PCR	1	3.85
**Assays for von Willebrand factor**		
ELISA	19	73.08
Bead assays	4	15.38
EIA	1	3.85
Immunoturbidimetry	1	3.85
Not specified	1	3.85
**Blood samples**		
Plasma	23	88.46
Serum	3	11.54

Abbreviations: PCR, polymerase chain reaction; EIA, enzyme immunoassay; RDT, rapid diagnostic test; ELISA, enzyme-linked immunosorbent assay.

**Table 2:** Subgroup analyses of vWF levels in *Plasmodium*-infected and uninfected participants.

Subgroup	Test for Subgroup Differences (Random-Effects Model)	SMD (95% CI)	*I*^2^ (%)	Number of Studies
**Publication years**	0.3088			
- 2020–2024		2.5376 [0.6354; 4.4398]	98.8	5
- 2010–2019		3.3182 [0.6293; 6.0071]	98.4	5
- 2000–2009		1.5756 [1.0488; 2.1024]	66.1	2
**Study design**	0.0602			
- Cohort studies		3.8231 [1.7725; 5.8737]	97.8	6
- Cross-sectional studies		1.5275 [0.2926; 2.7625]	96.6	6
**Continent**	<0.0001			
- Africa		1.3580 [0.7442; 1.9719]	97.6	8
- Asia		6.2783 [2.0879; 10.4686]	97.9	2
- South America		4.6809 [3.7558; 5.6060]	0.0	2
**Country**	<0.0001			
- Uganda		2.2526 [1.2978; 3.2074]	90.5	2
- Malawi		1.1840 [0.8093; 1.5588]	64.0	2
- Ghana		0.7092 [−1.4239; 2.8422]	99.1	2
- Nigeria		1.3662 [1.2137; 1.5187]	N/A	1
- Gabon		1.2505 [0.7000; 1.8009]	N/A	1
- Malaysia		6.2783 [2.0879; 10.4686]	97.9	2
- Brazil		4.6809 [3.7558; 5.6060]	0.0	2
**Age ranges**	0.0002			
- Adults		2.1086 [−2.8482; 7.0654]	98.2	2
- Children		1.6678 [1.1845; 2.1511]	95.8	6
- All age ranges		4.5980 [0.5205; 8.6755]	98.7	3
- Not specified		4.6819 [3.3734; 5.9903]	97.9	1
***Plasmodium* species**	0.0002			
- *P. falciparum*		1.3580 [0.7442; 1.9719]	97.6	8
- *P. vivax*		4.6809 [3.7558; 5.6060]	0.0	2
- *P. knowlesi*		8.4427 [7.3633; 9.5221]	N/A	1
- *P. falciparum*, *P. vivax*		4.1665 [3.6180; 4.7149]	N/A	1
**Diagnostic method for malaria**	0.0030			
- Microscopy		1.4942 [1.1735; 1.8150]	71.5	3
- Microscopy/RDT		0.6690 [−0.3838; 1.7217]	97.2	3
- Microscopy/PCR		4.3629 [2.5153; 6.2104]	96.8	6
**Methods for vWF**	0.0269			
- ELISA		2.5200 [1.1217; 3.9184]	98.2	11
- Bead assays		4.6819 [3.3734; 5.9903]	N/A	1
**Blood samples for vWF**	0.0583			
- Plasma		3.0990 [1.6563; 4.5416]	97.7	10
- Serum		0.6629 [−1.4049; 2.7306]	97.8	2

Abbreviations: RDT, rapid diagnostic test; CI, confidence interval; SMD, standardized mean difference; N/A, not assessed; vWF, von Willebrand Factor; PCR, polymerase chain reaction.

**Table 3:** Subgroup analyses of vWF levels in participants with severe and less severe *Plasmodium* infections.

Subgroup	Test for Subgroup Differences (Random-Effects Model)	SMD (95% CI)	*I*^2^ (%)	Number of Studies
**Publication years**	<0.0001			
- 2020–2024		12.3712 [11.4657; 13.2767]	N/A	1
- 2010–2019		2.6492 [−0.7446; 6.0429]	98.5	6
- 2000–2009		0.1348 [−0.2207; 0.4903]	N/A	1
**Study design**	0.0268			
- Cohort studies		0.3398 [0.1195; 0.5601]	25.5	4
- Cross-sectional studies		6.7968 [1.0861; 12.5075	99.5	4
**Continent**	0.0591			
- Africa		4.6404 [0.1582; 9.1227]	99.4	6
- Asia		0.3143 [0.0251; 0.6035]	0.0	2
**Country**	<0.0001			
- Malawi		4.5730 [−2.0400; 11.1859]	99.	3
- Malaysia		0.3143 [ 0.0251; 0.6035]	0.0	2
- Ghana		0.1348 [−0.2207; 0.4903]	N/A	1
- Nigeria		12.3712 [11.4657; 13.2767]	N/A	1
- Uganda		1.6668 [ 1.3136; 2.0201]	N/A	1
**Age ranges**	0.0591			
- Children		4.6404 [0.1582; 9.1227]	99.4	6
- All age ranges		0.3143 [0.0251; 0.6035]	0.0	2
***Plasmodium* species**	0.1072			
- *P. falciparum*		4.6404 [ 0.1582; 9.1227]	99.4	6
- *P. knowlesi*		0.1612 [−0.2671; 0.5895]	N/A	1
- *P. falciparum*, *P. vivax*		0.4426 [ 0.0505; 0.8347]	N/A	1
**Diagnostic method for malaria**	0.3689			
- Microscopy		4.7922 [−2.6849; 12.2694]	99.7	3
- Microscopy/RDT		5.9259 [−4.6111; 16.4630]	99.7	2
- Microscopy/PCR		0.7628 [−0.1470; 1.6726]	94.2	3
**Methods for vWF**	N/A			
- ELISA		3.5506 [−0.0074; 7.1085]	99.3	8
**Blood samples for vWF**	0.3005			
- Plasma		3.8232 [−0.2439; 7.8903]	99.4	7
- Serum		1.6668 [ 1.3136; 2.0201]	N/A	1

Abbreviations: RDT, rapid diagnostic test; CI, confidence interval; SMD, standardized mean difference; N/A, not assessed; vWF, von Willebrand Factor; PCR, polymerase chain reaction.

## Data Availability

All data relating to the present study are available in this manuscript, Table S1, Table S2, and Table S3 files.

## Supplementary Materials

The following supporting information can be downloaded at: https://www.mdpi.com/article/10.3390/medicina61040767/s1, Table S1: search terms; Table S2: details of included studies 4-09-2024; and Table S3: methodological quality of included studies_15-4-2025.
