# Supplementary material for: Alterations in von Willebrand Factor Levels in Patients with Malaria: A Systematic Review and Meta-Analysis of Disease Severity
Source: Medicina (Kaunas). 2025 Apr 21;61(4):767. doi: 10.3390/medicina61040767 (PMC12028635; doi:10.3390/medicina61040767)
Supplement: Supplementary file 1 [file medicina-61-00767-s001.zip › Table S1. Search terms.pdf]

Table S1. Search terms

General keywords

(“Von Willebrand factor” OR vWF OR “Factor VIIIR-Ag” OR “Factor VIIIR Ag” OR “Factor VIIIR-RCo” OR “Factor VIIIR RCo” OR “Factor VIII-Related Antigen” OR “Factor VIII Related Antigen” OR “Ristocetin-Willebrand Factor” OR “Ristocetin Willebrand Factor” OR “von Willebrand Protein” OR “Ristocetin Cofactor” OR “Plasma Factor VIII Complex”) AND (malaria OR plasmodium OR “Plasmodium Infection“ OR “Remittent Fever“ OR “Marsh Fever“ OR Paludism)

PubMed 13 June 2024

No.	Key concept	Search terms	Results
1.	Von Willebrand factor	“Von Willebrand factor”[MeSH Terms] OR “Von Willebrand factor”[Text Word] OR vWF[Text Word] OR “Factor VIIIR-Ag” [Text Word] OR “Factor VIIIR Ag”[Text Word] OR “Factor VIIIR-RCo” [Text Word] OR “Factor VIIIR RCo”[Text Word] OR “Factor VIII-Related Antigen”[Text Word] OR “Factor VIII Related Antigen”[Text Word] OR “Ristocetin-Willebrand Factor”[Text Word] OR “Ristocetin Willebrand Factor”[Text Word] OR “von Willebrand Protein”[Text Word] OR “Ristocetin Cofactor”[Text Word] OR “Plasma Factor VIII Complex”[Text Word]	25,825
2.	Malaria	malaria[Text Word] OR malaria[MeSH Terms] OR plasmodium[Text Word] OR “Plasmodium Infection“[Text Word] OR “Remittent Fever“[Text Word] OR “Marsh Fever“[Text Word] OR Paludism[Text Word]	122,636
3.	#1 AND #2	(“Von Willebrand factor”[MeSH Terms] OR “Von Willebrand factor”[Text Word] OR vWF[Text Word] OR “Factor VIIIR-Ag” [Text Word] OR “Factor VIIIR Ag”[Text Word] OR “Factor VIIIR-RCo” [Text Word] OR “Factor VIIIR RCo”[Text Word] OR “Factor VIII-Related Antigen”[Text Word] OR “Factor VIII Related Antigen”[Text Word] OR “Ristocetin-Willebrand Factor”[Text Word] OR “Ristocetin Willebrand Factor”[Text Word] OR “von Willebrand Protein”[Text Word] OR “Ristocetin Cofactor”[Text Word] OR “Plasma Factor VIII Complex”[Text Word]) AND (malaria[Text Word] OR malaria[MeSH Terms] OR plasmodium[Text Word] OR “Plasmodium Infection“[Text Word] OR “Remittent Fever“[Text Word] OR “Marsh Fever“[Text Word] OR Paludism[Text Word])	97

Embase 13 June 2024

No.	Key concept	Search terms	Results
1.	Von Willebrand factor	“Von Willebrand factor”/exp OR “Von Willebrand factor”:ti,ab,kw,de OR vWF:ti,ab,kw,de OR “Factor VIIIR-Ag”:ti,ab,kw,de OR “Factor VIIIR Ag”:ti,ab,kw,de OR “Factor VIIIR-RCo”:ti,ab,kw,de OR “Factor VIIIR RCo”:ti,ab,kw,de OR “Factor VIII-Related Antigen”:ti,ab,kw,de OR “Factor VIII Related Antigen”:ti,ab,kw,de OR “Ristocetin-Willebrand Factor”:ti,ab,kw,de OR “Ristocetin Willebrand Factor”:ti,ab,kw,de OR “von Willebrand Protein”:ti,ab,kw,de OR “Ristocetin Cofactor”:ti,ab,kw,de OR “Plasma Factor VIII Complex”:ti,ab,kw,de	50,914
2.	Malaria	malaria:ti,ab,kw,de OR plasmodium:ti,ab,kw,de OR ‘Remittent Fever’:ti,ab,kw,de OR ‘Marsh Fever’:ti,ab,kw,de OR Paludism:ti,ab,kw,de OR malaria/exp	162,444
3.	#1 AND #2	(“Von Willebrand factor”/exp OR “Von Willebrand factor”:ti,ab,kw,de OR vWF:ti,ab,kw,de OR “Factor VIIIR-Ag”:ti,ab,kw,de OR “Factor VIIIR Ag”:ti,ab,kw,de OR “Factor VIIIR-RCo”:ti,ab,kw,de OR “Factor VIIIR RCo”:ti,ab,kw,de OR “Factor VIII-Related Antigen”:ti,ab,kw,de OR “Factor VIII Related Antigen”:ti,ab,kw,de OR “Ristocetin-Willebrand Factor”:ti,ab,kw,de OR “Ristocetin Willebrand Factor”:ti,ab,kw,de OR “von Willebrand Protein”:ti,ab,kw,de OR “Ristocetin Cofactor”:ti,ab,kw,de OR “Plasma Factor VIII Complex”:ti,ab,kw,de) AND (malaria:ti,ab,kw,de OR plasmodium:ti,ab,kw,de OR ‘Remittent Fever’:ti,ab,kw,de OR ‘Marsh Fever’:ti,ab,kw,de OR Paludism:ti,ab,kw,de OR malaria/exp)	217

Scopus 13 June 2024

No.	Key concept	Search terms	Results
1.	Von Willebrand factor	TITLE-ABS-KEY (“Von Willebrand factor” OR vWF OR “Factor VIIIR-Ag” OR “Factor VIIIR Ag” OR “Factor VIIIR-RCo” OR “Factor VIIIR RCo” OR “Factor VIII-Related Antigen” OR “Factor VIII Related Antigen” OR “Ristocetin-Willebrand Factor” OR “Ristocetin Willebrand Factor” OR “von Willebrand Protein” OR “Ristocetin Cofactor” OR “Plasma Factor VIII Complex”)	39,538
2.	Malaria	TITLE-ABS-KEY (malaria OR plasmodium OR "plasmodium infection" OR "remittent fever" OR "marsh fever" OR paludism)	163,839

3.	1 AND 2	(TITLE-ABS-KEY ("Von Willebrand factor" OR vwf OR "Factor VIIIR-Ag" OR "Factor VIIIR Ag" OR "Factor VIIIR-RCo" OR "Factor VIIIR RCo" OR "Factor VIII-Related Antigen" OR "Factor VIII Related Antigen" OR "Ristocetin-Willebrand Factor" OR "Ristocetin Willebrand Factor" OR "von Willebrand Protein" OR "Ristocetin Cofactor" OR "Plasma Factor VIII Complex")) AND (TITLE-ABS-KEY (malaria OR plasmodium OR "plasmodium infection" OR "remittent fever" OR "marsh fever" OR paludis))	175

Ovid 13 June 2024

No.	Key concept	Search terms	Results
1.	Von Willebrand factor AND Malaria	("Von Willebrand factor" OR vWF OR "Factor VIIIR-Ag" OR "Factor VIIIR Ag" OR "Factor VIIIR-RCo" OR "Factor VIIIR RCo" OR "Factor VIII-Related Antigen" OR "Factor VIII Related Antigen" OR "Ristocetin-Willebrand Factor" OR "Ristocetin Willebrand Factor" OR "von Willebrand Protein" OR "Ristocetin Cofactor" OR "Plasma Factor VIII Complex") AND (malaria OR plasmodium OR "Plasmodium Infection" OR "Remittent Fever" OR "Marsh Fever" OR Paludism) {Including Limited Related Terms}	246

MEDLINE 13 June 2024

No.	Key concept	Search terms	Results
1.	Von Willebrand factor AND Malaria	(“Von Willebrand factor” OR vWF OR “Factor VIIIR-Ag” OR “Factor VIIIR Ag” OR “Factor VIIIR-RCo” OR “Factor VIIIR RCo” OR “Factor VIII-Related Antigen” OR “Factor VIII Related Antigen” OR “Ristocetin-Willebrand Factor” OR “Ristocetin Willebrand Factor” OR “von Willebrand Protein” OR “Ristocetin Cofactor” OR “Plasma Factor VIII Complex”) AND (malaria OR plasmodium OR “Plasmodium Infection“ OR “Remittent Fever“ OR “Marsh Fever“ OR Paludism)	109

ProQuest 13 June 2024

No.	Key concept	Search terms	Results
1.	Von Willebrand factor AND Malaria	(“Von Willebrand factor” OR vWF OR “Factor VIIIR-Ag” OR “Factor VIIIR Ag” OR “Factor VIIIR-RCo” OR “Factor VIIIR RCo” OR “Factor VIII-Related Antigen” OR “Factor VIII Related	803

		Antigen” OR “Ristocetin-Willebrand Factor” OR “Ristocetin Willebrand Factor” OR “von Willebrand Protein” OR “Ristocetin Cofactor” OR “Plasma Factor VIII Complex”) AND (malaria OR plasmodium OR “Plasmodium Infection“ OR “Remittent Fever“ OR “Marsh Fever“ OR Paludism)	

Google Scholar 13 June 2024

No.	Key concept	Search terms	Results
1.	Von Willebrand factor AND Malaria	Von Willebrand factor AND Malaria	Screening only the first 200 articles (all records, 6,100)
